# Transcriptomic and functional analysis of the *Anopheles gambiae *salivary gland in relation to blood feeding

**DOI:** 10.1186/1471-2164-11-566

**Published:** 2010-10-14

**Authors:** Suchismita Das, Andrea Radtke, Young-Jun Choi, Antonio M Mendes, Jesus G Valenzuela, George Dimopoulos

**Affiliations:** 1 W. Harry Feinstone Department of Molecular Microbiology and Immunology, Bloomberg School of Public Health, Johns Hopkins University, 615 N Wolfe Street, Baltimore, MD 21205-2179, USA; 2 Department of Pathobiological Sciences, University of Wisconsin-Madison, 1656 Linden Drive, Madison, WI 53706, USA; 3 Imperial College London, Division of Cell and Molecular Biology, Faculty of Natural Sciences, South Kensington Campus, London, UK; 4 Laboratory of Malaria and Vector Research, NIAID, National Institutes of Health, Rockville, Maryland 20852, USA

## Abstract

**Background:**

The *Anopheles gambiae *salivary glands play a major role in malaria transmission and express a variety of bioactive components that facilitate blood-feeding by preventing platelet aggregation, blood clotting, vasodilatation, and inflammatory and other reactions at the probing site on the vertebrate host.

**Results:**

We have performed a global transcriptome analysis of the *A. gambiae *salivary gland response to blood-feeding, to identify candidate genes that are involved in hematophagy. A total of 4,978 genes were found to be transcribed in this tissue. A comparison of salivary gland transcriptomes prior to and after blood-feeding identified 52 and 41 transcripts that were significantly up-regulated and down-regulated, respectively. Ten genes were further selected to assess their role in the blood-feeding process using RNAi-mediated gene silencing methodology. Depletion of the salivary gland genes encoding *D7L2*, *anophelin*, *peroxidase*, the *SG2 precursor*, and a *5'nucleotidase *gene significantly increased probing time of *A. gambiae *mosquitoes and thereby their capacity to blood-feed.

**Conclusions:**

The salivary gland transcriptome comprises approximately 38% of the total mosquito transcriptome and a small proportion of it is dynamically changing already at two hours in response to blood feeding. A better understanding of the salivary gland transcriptome and its function can contribute to the development of pathogen transmission control strategies and the identification of medically relevant bioactive compounds.

## Background

Adult mosquitoes feed on sugar to obtain energy for flight and other activities, while anautogenous females need a blood meal to develop eggs. Salivary glands and the saliva of insect disease vectors have attracted considerable attention because of their role in pathogen transmission and their production of pharmacologically active factors [1-4].

It is during the blood-feeding process that the *Plasmodium *parasite is taken up from an infected *A. gambiae *host. Once inside the mosquito, *Plasmodium *undergoes several developmental transitions and eventually becomes a sporozoite, which invades the salivary glands. This invasion represents a critical step in the transmission of the parasite to the vertebrate host. Completion of the infection depends on the injection of sporozoites, through the saliva, into the host's skin and leaving the inoculation site rapidly to enter and invade the liver for further development [5,6].

Unlike male salivary glands, female mosquito salivary glands possess anti-hemostatic, vasodilatory and immune-modulatory factors to facilitate the acquisition of blood, while salivary glands of both sexes have activity related to the digestion of the sugar meal as well as antimicrobials to prevent microbial growth [7,8]. Mosquitoes have been shown to require longer probing times during blood-feeding when an apyrase gene (an enzyme that counteracts hemostasis) has been silenced, or if they are deprived of salivation by removal of the salivary duct through which the saliva is transported to the probing site [9,10]. Silencing of another *A. gambiae *salivary gland gene, *SG6 *(a small protein with unknown function), results in increased probing time and reduced blood-feeding ability [11]. Previous studies have shown that several *Anopheles *salivary gland proteins are reduced after blood-feeding, suggesting that these major polypeptides may have been introduced into the vertebrate hosts during the blood meal [12,13]. There is evidence that the pharmacological activity of arthropod saliva affects pathogen transmission and the local inflammatory response of the host. For example, the salivary gland lysate from the sand fly *Lutzomyia longipalpis *facilitates the infection of mice by the protozoan parasite *Leishmania major *[14,15]. It has also been shown that the *L. longipalpis *salivary gland lysate inhibits neutrophil migration and the Th1 immune inflammatory response. These findings suggest that the compounds responsible for such activities could be used for the development of novel anti-inflammatory drugs [4].

While earlier sialo-transcriptomic studies have identified a variety of salivary gland genes, [1,16-21], we present the first global microarray transcriptome analysis of the *A. gambiae *salivary gland under conditions related to feeding. Earlier studies have identified some 3,000 adult female *A. gambiae *salivary gland-transcribed sequence tags and 4,719 genes were found to be transcribed in the larval gland [22], of which 747 were specific for this tissue. Here we report 4,978 adult female *A. gambiae *salivary gland transcripts, as defined by oligonucleotide microarray gene transcription analysis. We show that 52 and 41 salivary gland-expressed transcripts were up-regulated and down-regulated, respectively, at 2 hours after blood-feeding when compared to salivary glands of unfed mosquitoes. We have also used an RNAi-mediated gene silencing approach to assess the potential involvement of 10 selected salivary gland genes in regulating mosquito blood-feeding capacity. Silencing of several salivary gland transcripts; *D7L2*, *anophelin*, *peroxidase*, *5'nucleotidase *and *SG2 precursor*, produced a significantly lowered blood-feeding phenotype and increased probing time, confirming that these genes may be playing an important role in blood-feeding. The updated list of the *A. gambiae *salivary gland transcriptome, together with the results of our comprehensive functional analyses, can facilitate the discovery of novel pharmacologically active compounds and provide tools for the development of malaria control strategies.

## Results and Discussion

### The *A. gambiae *salivary gland transcriptome

**Figure 1:**
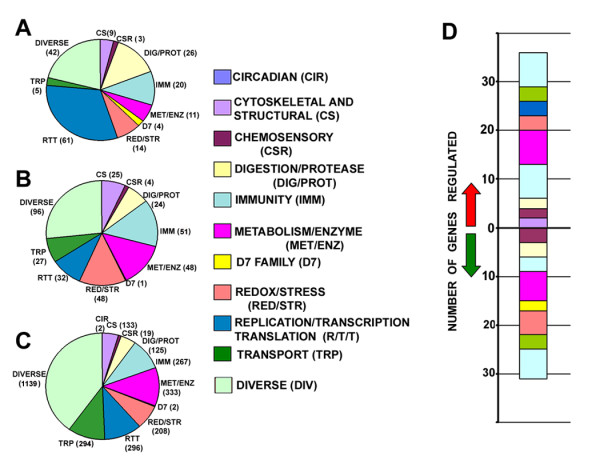
**The adult female *A. gambiae *salivary gland transcriptome and its regulation upon blood-feeding**. Genes that correspond to replica spots with a mean salivary gland RNA probe fluorescence hybridization intensity value >100 were grouped into the following three categories: **(A) **highly abundant transcripts (n = 267), with fluorescence values from 5,000 to 65,000; **(B) **moderately present transcripts (n = 482), with fluorescence values from 1,000 to 4,999; and **(C) **weakly present transcripts (n = 4,229), with fluorescence values from 100 to 999. The three categories were further sub-grouped into 11 functional groups according to their predicted functions, as shown in the pie charts. **(D) **The bar graph shows the number of genes that were regulated upon blood-feeding in different functional groups; those that were up-regulated (52 genes) are above zero (indicated by a red arrow), and those that were down-regulated (41 genes) are below zero (indicated by a green arrow). The color code for each group is shown. Those genes belonging to the unknown functional group are not shown in any of the diagrams.

In order to characterize the *A. gambiae *female salivary gland transcriptome, we employed a microarray-based genome transcription approach to compare the transcript abundance in the salivary glands at 2 hours after blood feeding to salivary glands of unfed female mosquitoes. The relative transcript abundance of salivary gland expressed genes was analyzed by sorting the blood-fed versus unfed salivary gland transcripts into three different categories based on their corresponding spot fluorescence intensities (Figure 1A,B,C; see Additional file 1). Of the total predicted transcriptome (13,133 genes of *A. gambiae*, according to version 48, ENSEMBL); 4,978 genes (38%) genes were found to be transcribed in the salivary gland of 4-day-old *A. gambiae *female mosquitoes. Of these, 267 transcripts (2.1%) were considered as highly abundant (with fluorescence values ranging from 5,000 to the maximum threshold of 65,000; Figure 1A and Sheet 1 of Additional file 1); 482 transcripts (3.6%) were considered as moderately present (fluorescence values ranging from 1,000 to 4,999; Figure 1B and Sheet 2 of Additional file 1), and 4,229 transcripts (32.2%) were considered as being present at low levels based on spot intensity (fluorescence values ranging from 100 to 999; Figure 1C and Sheet 3 of Additional file 1).

We analyzed the predicted functional class composition of each category (discussed below) to gain insight into the major processes that take place in the female mosquito's salivary glands upon blood-feeding.

#### Cytoskeletal and structural genes

Several cytoskeletal and structural gene transcripts (9 highly, 25 moderately, and 133 weakly transcribed) were identified in the salivary glands (Figure 1A, B, C and see Additional file 1). One of the most abundant transcripts encoded a mucin, a secretory protein that has been shown to be a major protein in the salivary gland lysate of several mosquito species [1,16,19]. This protein may function as a lubricant of the mosquito mouthparts and has been proposed to bind to the chitinous linings of the salivary ducts and mouthparts [1,23]. Three mucin encoding transcripts have been identified in the *A. gambiae *larval salivary gland [22], suggesting the importance of mucins at multiple developmental stages. We have further investigated the function of one of the mucin gene (AGAP001192-RA) by assaying changes in the feeding and probing behavior of mosquitoes after its depletion through RNAi (discussed below). In addition, *Saglin *(AGAP000610-RA) was shown to be an abundant transcript in the salivary gland. Saglin encodes for a secretory protein (100 kDa) that has been shown to bind to the *Plasmodium berghei *sporozoite protein thrombospondin related anonymous protein (TRAP), and play a crucial role in salivary gland invasion by sporozoites, [24,25]. The presence of transcripts such as *actin*, *chitin binding*, *cuticlar protein*, *myosin*, *laminin*, *tropomysin*, *tubulin*, *annexin*, *dynein light chain*, *connectin*, *dynactin*, *cadherin*, and *myosin muscle *suggests that these genes play a significant housekeeping role in the *A. gambiae *salivary gland.

#### Genes involved in protein and sugar digestion

As many as 175 genes putatively involved in protein and sugar digestion were found to be transcribed in the *A. gambiae *salivary glands; 26 of these transcripts were highly abundant, 24 were moderately present and 125 were present at low levels (Additional file1). Many of these transcripts coding for proteases (*peptidase C1*, *peptidase S1*, *peptidase S6, TRY2*, *TRY3*, *TRY4*, *TRY5*, *TRY6*, *TRY7*, several *serine proteases*, *carboxypeptidases*, and *aminopeptidases*) and glycosidases (four *maltase *transcripts AGAP011939-RA, AGAP012401-RA, AGAP002102-RA, and AGAP008965-RA and a chitinase transcript AGAP008061-RA) have a secretory signal peptide sequence.

Salivary gland glycosidases have been shown to be involved in the digestion of sugar, whereas the proteolytic enzymes could be involved in host-specific proteolytic events (clot prevention or the complement and coagulation cascades, or in the digestion of extracellular matrix components) that occur during the blood-feeding process. It is also possible that these proteolytic enzymes, which are secreted in the saliva, are ingested along with the blood, and might be involved in its digestion. Proteins encoded by transcripts that lack signal peptide sequences are most likely involved in housekeeping functions in the salivary gland or may be secreted by an alternative non-classical method [26]. The majority of these genes are also transcribed in the *A. gambiae *midgut and other tissues as well as during other developmental stages as has been demonstrated in earlier studies [27-29]. The proteolytic digestive enzymes in the midgut are mainly involved in the digestion of blood.

#### Circadian and chemosensory genes

Two circadian system-related genes, *takeout *and *timeless*, were weakly transcribed in the salivary gland. *Takeout *has been shown to act as a molecular link between circadian rhythm and feeding behavior in *Drosophila melanogaster *and *A. gambiae *[30,31], and *timeless *is a transcription factor that plays a major role in circadian regulation [32]. The *timeless *transcript has been identified previously in the *A. gambiae *female salivary gland [1]; however, we report for the first time the expression of *takeout *in this tissue.

Several genes with putative chemosensory functions, such as *odorant binding proteins *(*OBPs*), *odorant receptors*, *antennal carrier protein*, *arrestins*, *gustatory receptors*, and *pheromone binding proteins*, were transcribed in the *A. gambiae *salivary gland (3 were highly expressed, 4 were moderately expressed, and 19 were weakly expressed; see Additional file 1). *OBPs *are mainly localized in the mosquito head and within the antennae, where they function as carriers of odorants to the olfactory receptors [33]. The D7 family of proteins are distantly related to the OBP super-family, because of the conserved 6-cysteine and 10-cysteine residues and a characteristic fold structure [34]. However, the OBPs function as odorant carriers [33], whereas D7 proteins have been proposed to inhibit hemostasis by trapping agonists of hemostasis [35], and thereby facilitate blood-feeding by hematophagous vectors (discussed in detail in the next section).

#### D7 protein family genes

The four highly transcribed members of the *A. gambiae *D7 family were the *D7r-1, 2, 3 *precursors (short form) and the *D7 long form L2 *(AGAP008279-RA) (fluorescence values above 5,000), whereas the D7r-4, 5 precursors (short form) and another *D7 long form L1 *(AGAP008278-RA) were found to be only moderately/weakly transcribed in the salivary glands (fluorescence values between 100 and 4,999) (see Additional file 1). The *D7 *gene (coding for salivary biogenic-amine binding protein) was first identified in *A. aegypti *[36] as a major salivary gland secretory protein. The D7 protein exists in two forms: a long form (~30-35 kDa), which is found exclusively in mosquitoes and sand-flies, and the short forms (~15 kDa), which are found in other insects [37]. Five different D7-related (D7r1, 2, 3, 4, and 5) short forms and three D7 long forms have been identified in the *A. gambiae *adult salivary gland [1,38]. In 2006, Calvo *et al. *proposed that D7-related proteins from blood-sucking insects act as anti-hemostatic factors by trapping agonists of hemostasis. One short D7 protein from *A. stephensi*, hamadarin (D7r1), has been shown to inhibit the plasma contact system by preventing the activation of kallikrein by Factor XIIa [39]. The D7r1, 2, 3, 4 and D7 long forms have been shown to bind to the biogenic amines serotonin, histamine, and norepinephrine [1,35]. The crystal structures of one member of the D7 family, D7r4 (short form), and those of the ligand complexes were determined in order to elucidate the mechanisms governing its anti-hemostatic and anti-inflammatory properties [34]. It has been suggested that the ancestral D7 has originated from the proteins of the OBP family, which were adapted to bind small hydrophobic ligands [34,40]. We have further investigated the function of this family by looking at the changes in feeding and probing behavior of mosquitoes after silencing of the two D7 long forms, *D7 L1 *and *D7 L2 *(discussed below).

#### Immunity- and stress-related genes

Transcripts representing 338 immune genes (20 highly transcribed, 51 moderately transcribed, and 267 weakly transcribed) were found to be expressed in the *A. gambiae *female salivary glands (see Additional file 1). These genes included: *lysozyme*, *gram negative bacteria binding protein *(*GNBP*), the anti-microbial peptides *cecropin *and *defensin*, the NFκB transcription factors *Rel1 *and *Rel2*, a *c-type lectin *(*CTL*), *galectins*, several *fibrinogen binding proteins *(*FBN*), *serpins*, *thioester containing proteins *(*TEP*), a *caspase*, *SOCS*, *TOLL*, *myd-88*, a *leucine rich receptor *(*LRR*), a *scavenger receptor*, a *prophenoloxidase *(*PPO*), and others. Immune genes such as *c-type lectins*, *prophenoloxidase activating enzymes*, *galectins*, *V5 allergens*, and *lysozyme *were identified in the *A. gambiae *salivary gland [1,16,19] and in another study, it was shown that *GNBP*, *defensin*, putative *gal-lectin*, and putative *serine protease *transcripts were up-regulated in the *A. gambiae *salivary gland after *Plasmodium berghei *infection [41]. In a more recent SAGE analysis of the *A. gambiae *salivary gland transcriptome upon *P. berghei *infection; 37 immune related genes were identified and four of them (d*efensin1*, *GNBP*, *serpin*, *cecropin2*) were up-regulated during salivary gland invasion by the sporozoites [42].

Several of these immune genes encode secretory proteins, including GNBPs, several FBNs, the short antimicrobial peptides, PGRPs, chitinase, and lectins. Till date, little is known about the role of these immune genes in the *A. gambiae *female salivary gland, or whether they are directly involved in anti-*Plasmodium *or anti-microbial defenses. The secretory immune proteins may also be involved in protective immunity against microbes or other pathogens with which the mosquitoes come into direct contact during feeding. In the larval salivary gland, three *defensins*, three *fibrinogen binding proteins*, one *CLIPB8*, and four *serine proteases *have been detected [22]. Three different *chitinase *transcripts have been detected in *A. gambiae *larval salivary glands [22] and have been proposed to protect against pathogenic bacteria and fungi derived from the mosquitoes' diet [43]. Of the three different *lysozyme *transcripts detected in this study, one (AGAP007386-RA) was also detected in the salivary gland of larvae [22]. Lysozyme might be involved in preventing the growth of microbes in the sugar meal that is ingested with saliva and stored in the mosquito crop [44]. Most of these immune genes are also transcribed in other tissues, where they are involved in limiting bacterial and parasite growth [29,45-49]. Warr and coworkers [29] have shown that the cardia and anterior part of the midgut transcribe most of the antimicrobial peptides and other anti-*Plasmodium *factors.

Among the genes involved in oxidoreductive, antioxidant, and stress-related processes in *A. gambiae*, 270 transcripts were found to be present in the salivary glands (14 highly transcribed, 48 moderately transcribed, and 208 weakly transcribed) (see Additional file 1). This group was composed of several *cytochrome P450 enzymes*, *glutathione s-transferase*, *cytochrome c oxidase*, *NADH ubiquinone*, *DNAJ homolog*, *oxidoreductase*, *animal heme peroxidase*, *thiorededoxin 1*, *mitochondrial inner membrane protein*, and many others. Some of these transcripts have been identified earlier in salivary gland EST libraries or in other studies of different mosquito species [1,16,19,50] and are involved in housekeeping functions of the salivary gland. Several transcripts coding for *heat-shock proteins *(*HSPs*) were found to be transcribed in the female salivary gland. HSPs belong to a family of proteins that function as chaperones or are involved in cellular defense against external stress from various sources [51].

#### Metabolism and other enzyme genes

As many as 392 transcripts (11 highly transcribed, 48 moderately transcribed, and 333 weakly transcribed) of genes putatively involved in metabolism and other enzymatic activities were found to be transcribed in the salivary gland of *A. gambiae *(see Additional file 1). Among the transcripts encoding putative secretory enzymes, three members of the 5' nucleotidase gene family; an *apyrase *precursor (AGAP011971-RA), and two *5' nucleotidase *precursors (AGAP011026-RA and AGAP003629-RA) were found to be transcribed in the adult salivary gland (see Additional file 1). The *apyrase *and one of the *5' nucleotidase precursors *(AGAP011026-RA) have been shown to be components of saliva [23,52]. The second *5' nucleotidase precursor *gene contains a secretory signal sequence, suggesting potential involvement in feeding. Of these three 5' nucleotidase gene family transcripts, the AGAP011026-RA was highly expressed, and the other two were moderately expressed. Enzymes of this family facilitate the acquisition of blood meals by removing and degrading pharmacologically active ATP/ADP nucleotides from the site of the injury [53].

Silencing of an *apyrase *gene in the salivary gland of *A. gambiae *resulted in an increase in the mosquito probing time as well as increase *in vitro *platelet aggregation [9]. We have further investigated the function of this gene family by assaying changes in the feeding and probing behavior of mosquitoes after silencing the *5' nucleotidase precursor *transcript (AGAP011026-RA) (discussed below). Some of the metabolic enzymes with a signal peptide sequence include alpha amylase (AGAP002317-RA) and alpha glucosidase (AGAP000862-RA), which are weakly transcribed in the female salivary glands. The transcript *glycohydrolase *(AGAP008124-RA), which is weakly transcribed in the *A. gambiae *salivary gland, also encodes a secretory protein and belongs to a large family with diverse functions reflecting their amino acid sequences: chitosanase, and NAD+ glycohydrolase, among many others [54].

#### Genes of other functional groups

Several genes involved in housekeeping functions and belonging to the replication-transcription-translation (389 genes in total) and transport functional groups (326 genes in total) were found to be transcribed in the *A. gambiae *salivary gland (see Additional file 1). In the replication-transcription-translation group, genes including several ribosomal proteins, histones, elongation factors, repressors, activators, proteasome subunits, and enzymes such as helicase, ligase, polymerase, DNA repair, ribonuclease, and many other enzymes were found to be transcribed. In the transport group, transcripts such as *vesicle transport protein*, *solute carrier protein*, *ion transporter*, *ion channels*, *apolipophorin precursor*, *aquaporin water channel*, and many others were found to be transcribed. Several transcripts (including *cyclin*, *ferritin*, *zeelin 1*, *anophelin*, *trio*, *zinc finger domain*, *SG2*, *SG3*, *SG5*, *SG6 *and *SG10*, many hypothetical precursors/peptides, and others) were found to be transcribed and were placed in the diverse group (1,277 transcripts in total) because of their assorted roles and various functions. The presence of vitellogenin precursor transcripts is likely to indicate contamination with fat body which is attached to the salivary glands.

A study recently showed that silencing of *SG6 *transcript results in an increased probing time and reduced feeding ability in *A. gambiae *female mosquitoes [11]. In this study we have investigated the function of the *SG2 *transcript (the function of the SG2 protein is as yet unknown), by assaying changes in the feeding and probing behavior of mosquitoes after its depletion (discussed below). The SG1 protein precursor, a secretory protein, was found to be highly transcribed in the salivary gland; SG1 protein family members have molecular masses around 44·kDa and do not exhibit significant similarities [55] to other proteins in the NCBI database, with the exception of the *Anopheline *distantly related TRIO protein (an abundant salivary gland gene) [3,19], when analyzed by BLAST analysis. Transcripts of unknown function or with no homology to known protein were assigned to the group of unknown (1,609 transcripts in total). Further studies are required to link these transcripts with specific roles of salivation, feeding, blood digestion, and storage in *A. gambiae*.

### Regulation of *A. gambiae *salivary gland transcripts upon blood-feeding

We have performed a microarray-based gene transcription analysis of the blood-feeding-regulated genes in the *A. gambiae *salivary gland. We compared the transcriptome of the *A. gambiae *salivary glands at 2 h after blood-feeding to salivary glands of unfed mosquitoes to identify salivary gland transcripts that are likely to play a role, either directly or indirectly, in the feeding process. Previous studies have shown that salivary gland proteins are rapidly depleted during feeding and that this depletion is proportional to the duration of feeding. Since blood fed mosquitoes are capable of taking a second blood-meal already at 24 hours after the first feeding, we hypothesized that the replenishment of the salivary gland proteins must initiate soon after feeding, and therefore selected a 2 hour time point after feeding to assay blood feeding-related changes in the salivary gland transcriptome [23,56]. Genome-wide microarray analysis of gene expression responses to blood feeding has been performed previously but for whole female *A. gambiae *mosquitoes [57], whereas our study is exclusively for the female salivary glands.

The resulting catalog of transcripts that exhibited differences in abundance prior and after blood feeding in the *A. gambiae *salivary gland is presented in Additional file 2. In total, 93 transcripts were differentially regulated; 52 transcripts were more abundant and 41 transcripts were less abundant after blood-feeding (Figure 1D and see Additional file 2). Marinotti *et al*. showed in 2005 that some 4,924 transcripts, derived from whole mosquitoes displayed changes in abundance within 24 hour after blood-meal; 2,388 genes were up-regulated while 2,536 genes were down-regulated [57]. The salivary gland expressed transcripts that were significantly regulated by blood-feeding are presented in Table 1 (excluding the diverse and unknown groups).

**Table 1:** The female *A. gambiae *salivary gland expressed genes that are significantly differentially regulated upon blood-feeding.

GENE NAME	TRANS-CRIPT ID	FUNCTIONAL GROUP	**Log**_**2 **_**TRANSFORMED VALUE (MEAN)**
STATHMIN	E013307	CS	1.20

CHITIN BINDING PROTEIN	E022689	CS	1.08

PHEROMONE BINDING PROTEIN	E013682	CSR	1.60

ODORANT BINDING PROTEIN	E017475	CSR	0.75

ODORANT BINDING PROTEIN	E021953	CSR	-0.79

ODORANT BINDING OBP10	E012102	CSR	-0.85

ODORANT BINDING OBP7	E012251	CSR	-0.90

TRYPSIN 2 PRECURSOR	E027737	DIG/PROT	1.11

PROTEASE PRECURSOR	E018532	DIG/PROT	0.80

AMINOPEPTIDASE N	E002729	DIG/PROT	-0.82

AMINOPEPTIDASE	E012865	DIG/PROT	-1.07

TRYPSIN 6 PRECURSOR	E018354	DIG/PROT	-1.09

D7 L2 PRECURSOR ALLERGEN AED A 2	E018280	D7 FAMILY	-1.75

D7 L1PRECURSOR ALLERGEN AED A 2	E023833	D7 FAMILY	-2.01

RELISH	E020147	IMMUNE	1.14

REL1	E011101	IMMUNE	0.90

CASPS5	E021365	IMMUNE	0.82

LYSOZYME c-7	E018439	IMMUNE	0.78

LECTIN	E010670	IMMUNE	-0.75

E1 PROTEIN Def2/Der2 ALLERGEN	E017011	IMMUNE	-0.80

CECROPIN B	E011995	IMMUNE	-0.96

DEFENSIN	E015621	IMMUNE	-1.04

ETHANOLAMINE PHOSPHATE TRANSFERASE	E012337	MET/ENZ	1.45

ELONGATION OF VERY LONG CHAIN FATTY ACIDS	E010068	MET/ENZ	1.40

L 3 PHOSPHOSERINE PHOSPHATASE	E016587	MET/ENZ	1.02

LIPOAMIDE ACYLTRANSFERASE	E024823	MET/ENZ	0.94

PYRIDOXAL PHOSPHATE PHOSPHATASE	E024098	MET/ENZ	0.89

PROTEIN KINASE	E012934	MET/ENZ	0.89

DIPEPTIDYL PEPTIDASE IV	E010468	MET/ENZ	0.75

GLYCEROL 3 PHOSPHATE DEHYDROGENASE	E017587	MET/ENZ	-0.79

ISOCITRATE DEHYDROGENASE [NAD] SUBUNIT	E010852	MET/ENZ	-0.80

SERINE PYRUVATE AMINOTRANSFERASE	E015996	MET/ENZ	-1.09

CYTOCHROME P450	E026706	REDOX/STRESS	1.57

CYTOCHROME P450	E029062	REDOX/STRESS	1.51

DNAJ	E022059	REDOX/STRESS	1.46

GLUTATHIONE S TRANSFERASE	E012678	REDOX/STRESS	-0.78

COX5B CYTOCHROME C OXIDASE	E011122	REDOX/STRESS	-0.86

MITOCHONDRIAL UBIQUINOL CYTOCHROME C	E017699	REDOX/STRESS	-0.92

UBIQUINOL CYTOCHROME C REDUCTASE	E021887	REDOX/STRESS	-0.97

OXIDOREDUCTASE	E021713	REDOX/STRESS	-1.04

CCR4 ASSOCIATED FACTOR 2	E010439	RTT	1.68

MEDIATOR OF RNA POLYMERASE II	E021242	RTT	1.49

SPLICING FACTOR ARGININE/SERINE RICH	E016046	RTT	1.27

SUCCINATE DEHYDROGENASE [UBIQUINONE]	E019261	TRP	1.60

VACUOLAR H+-TRANSPORTING C SUBUNIT	E013400	TRP	1.03

ADP ATP CARRIER ADP/ATP TRANSLOCASE	E020278	TRP	-0.87

ATP SYNTHASE B CHAIN MITOCHONDRIAL	E012069	TRP	-0.94

ADP ATP CARRIER ADP/ATP TRANSLOCASE	E014881	TRP	-1.70

The ENSEMBL transcript IDs have been shortened by replacing the first 12 characters of ENSANGT00000 with "E". The genes are sorted into different functional groups according to their predicted functions (CS: cytoskeletal and structural; CSR: chemosensory; PROT/DIG: proteolytic digestion; D7 family: D7 family; IMM: immunity; MET/ENZ: metabolism and other enzymes; RED/STR: redox/stress; R/T/T: replication/transcription and translation; TRP: transport). The mean log_2_-transformed-fold transcript abundance ratio is shown in column 4.

The down-regulation of several female *A. gambiae *salivary gland transcripts can be attributed to the depletion of transcripts during protein translation or to the degradation of transcripts following blood-feeding. It is possible that their transcription may have been shut down because these transcripts were no longer required until the next blood-meal. Down-regulation of several *A. aegypti *salivary gland transcripts upon blood-feeding has been shown in a recent publication; the list of transcripts involved coded for secretory proteins, such as odorant binding protein, protease inhibitors, immune genes and other [58].

The secretory protein encoding salivary gland transcripts that displayed a lower abundance after blood-feeding included two OBPs (OBP 10 and OBP 7), two *D7 long-form precursors (L1 and L2)*, two *aminopeptidases*, a *trypsin 6 precursor*, a *salivary lipase*, a *5' nucleotidase precursor*, an *apyrase*, *E1 protein*, *cecropin 3*, *defensin 1*, and a *hypothetical 6.2 precursor*. In earlier studies, several *OBP *transcripts were shown to be down-regulated after blood-feeding in the head and antennae of *A. gambiae *[30] and in the salivary gland of *A. aegypti *[58]. In Marinotti *et al.*, 2005, transcripts of several serine protease, aminopeptidase, odorant binding protein and transporter genes were found to be down-regulated in whole female mosquitoes 24 hrs after blood feeding. The OBP and odorant receptors are required by the female mosquitoes to sense host olfactory cues. The fact that the OBPs are not required after feeding until the next blood-meal may account for their lower transcript abundance. Both the secretory proteins aminopeptidases and trypsin 6 may be required for the digestion of host proteins or extracellular matrix components [59]. The apyrase and 5' nucleotidase proteins are known to facilitate the acquisition of a blood meal by removing pharmacologically active nucleotides that are important for platelet aggregation at the site of the injury [53]. The D7 proteins inhibit hemostasis, and one short *A. stephensi *D7 protein, hamadarin, has been shown to inhibit the plasma contact system [39]. The secretory immune proteins like *cecropin *and *defensin *may provide protection against microbes or other pathogens that the mosquitoes encounter during feeding. In 2007, Rosinski-Chupin *et al. *showed that the immune peptide *defensin1 *and *cecropin 2 *transcripts were up-regulated in *P. berghei *infected *A. gambiae *salivary glands, thereby demonstrating that the salivary epithelium responds to the presence of pathogens [42].

Several transcripts were up-regulated after blood-feeding, and those that encode putative secretory proteins included a t*rypsin 2 precursor*, *lysozyme*, an *OBP*, an *ATP binding protein*, a *vitellogenin precursor*, and a *DNAJ precursor*. In contrast, two lysozyme transcripts were found to be down-regulated in whole female *A. gambiae *mosquitoes at 24 hours after blood-meal [57]. Lysozyme has been proposed to prevent bacterial growth in the sugar and blood meals [1,16]. The DNAJ family of proteins is involved in protein folding, protein transport, and the cellular response to stress [60]. The exact role of these genes in the salivary gland and their function in the blood-feeding process are still not clear. We have further investigated the function of some of these differentially regulated genes by looking at how they influence the mosquito feeding and probing behavior upon depletion.

### Functional analysis of salivary gland gene implication in blood-feeding and probing behavior

**Figure 2:**
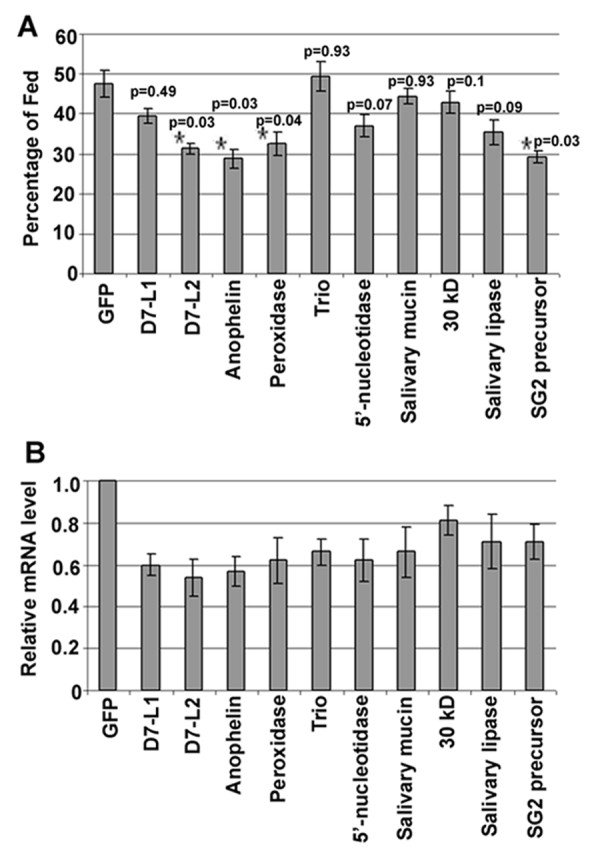
**Implication of salivary gland genes in blood-feeding capacity**. **A**. The percentage of gene-silenced mosquitoes that fed on a mouse after gene-specific silencing through RNAi compared to the GFP dsRNA injected control mosquitoes. Data were pooled from six replicates of 25 mosquitoes each. Genes displaying significant capacity to alter blood feeding propensity upon silencing are indicated with an asterisk, and the p-values are shown for each gene (from the Mann-Whitney test). The error bars indicate the standard error. **B**. Determination of RNAi-mediated gene silencing efficiency, by qRT-PCR at 4 days after dsRNA injection. The cDNA templates were normalized using the AgS7 gene specific primers. The graph shows the relative transcript abundance of each gene after knockdown, as compared to its abundance in the GFP dsRNA-treated control mosquitoes (set to 1.0). The error bars indicate the standard error.

To identify putative components of the *A. gambiae *salivary gland that play a role in blood-feeding, we performed transient RNAi gene-silencing assays on 10 salivary gland transcribed genes and studied the effect of this treatment on mosquito feeding behavior on a vertebrate host (Figure 2A). In our first assay, instead of assaying probing time of individual mosquitoes we determined the blood feeding capacity (4 days after dsRNA injections) as a measure of their feeding status after 20 minutes exposure to the vertebrate host since probing time may not be the only determining factor of feeding capacity, nor an exclusive indicator for a salivary gland function that facilitates feeding. It is conceivable to hypothesize that the amount of ingested blood is dependent on probing time, feeding time and the flow rate of blood which may depend on its viscosity. We specifically compared blood-feeding capacity between gene silenced and control group mosquitoes that had been injected with GFP dsRNA according to previously established methodology [30]. Four of these genes, two *D7 long forms *(*D7L1 *and *D7L2*), one *salivary gland peroxidase 5B *(AGAP010735-RA), and one *5' nucleotidase *(AGAP011026-RA), were selected because of their predicted anti-inflammatory, vasodilatory, and anti-platelet activities, respectively [39,53,61] as well as their down-regulation after blood-feeding (see Additional file 2). A salivary gland lipase enzyme (AGAP005822-RA) whose function is not clear and down-regulated after blood-feeding (see Additional file 2) was selected for functional analysis. Five other genes were selected based on their predicted involvement in anti-hemostatic processes and for representing major *A. gambiae *salivary gland secretory proteins: *anophelin/cEF *(AGAP008004-RA), which acts as an α-thrombin inhibitor to prevent blood coagulation [62]; a *salivary 30 kDa protein *(AGAP009974-RA), a member of the aegyptin family, and whose members have been shown to bind to collagen and block platelet adhesion to collagen [63,64]; an *SG2 precursor *(AGAP006506-RA) with unknown function; and *salivary mucin *(AGAP001192-RA) and *TRIO *(AGAP001374-RA), which are two highly abundant salivary gland transcripts in *A. gambiae *[1,19,62] (see Additional file 1). In *Drosophila melanogaster*, the TRIO protein has been shown to be involved in the cytoskeletal rearrangements necessary for cell migration [65]; however, its exact function in *A. gambiae *is not known.

RNAi-mediated gene silencing assays in adult mosquitoes depend on the direct injection of dsRNAs into the hemolymph and we used the dsRNA injection procedure established by Boisson *et al.*, 2006 (see Materials and Methods) and monitored gene silencing efficiency in the salivary glands 4 days after dsRNA injections (Figure 2B). Silencing efficiency for the selected genes ranged from 19% to 46% (Figure 2B and see Additional file 3). The effect of gene silencing on the mosquito's blood-feeding capacity is shown in Figure 2A, and the statistical analysis of the data is presented in Additional file 4.

Only mosquitoes in which *D7L2*, *anophelin*, *peroxidase 5B*, or *SG2 precursor *was silenced displayed a significantly altered feeding capacity (by ~15-22%) when compared to the GFP dsRNA-injected control mosquitoes (Figure 2A). The D7 proteins have been shown to bind biogenic amines such as serotonin and histamine, which may antagonize the vasoconstriction, platelet aggregation, and pain induction that occur during feeding [34]. The lower feeding capacity observed after the silencing of *D7L2 *strongly supports the involvement of its encoded protein (and of other members of this family) in the blood-feeding process. This protein may act as an anti-hemostatic factor by trapping agonists of hemostasis, as suggested by Calvo *et al. *(2006). The salivary peroxidase 5B is known to have vasodilatory activity. Therefore, we hypothesize that the lower blood-feeding capacity of peroxidase-silenced mosquitoes is due to the resulting increased vasoconstriction and diminished blood-flow at the probing site. The *anophelin *gene encodes an α-thrombin inhibitor that prevents blood coagulation during the blood-feeding process. Depletion of the *anophelin *protein is expected to decrease the blood-flow at the site of probing; an effect that may account for the lower feeding propensity of the *anophelin*-silenced mosquitoes. Silencing of a small secretory peptide SG2 precursor also resulted in a decreased blood-feeding capacity, although its role during blood-feeding is not yet clear. In earlier studies, silencing of a salivary *apyrase *gene (involved in the inhibition of platelet aggregation in *A. gambiae*) and a SG6 precursor led to an increased probing time and decreased blood-feeding ability in *A. gambiae *mosquitoes [9,11]. However, further studies are needed to characterize the biological properties of this unknown peptide SG2.

The *D7L1 *and *5' nucleotidase *genes are known to have anti-inflammatory and anti-platelet activity, respectively; however, the silencing of these two genes resulted in only an 8-10% decrease in their blood-feeding capacity (when compared to the GFP control). The lack of any significant effects on feeding after silencing of the remaining six genes (*D7L1*, *TRIO*, *5' nucleotidase, salivary mucin*, *salivary lipase*, and *30 kDa*) may be related to the relatively low silencing efficiency obtained in the salivary gland as well as to the presence of unknown factors that perform redundant functions in the mosquito salivary gland.

**Figure 3:**
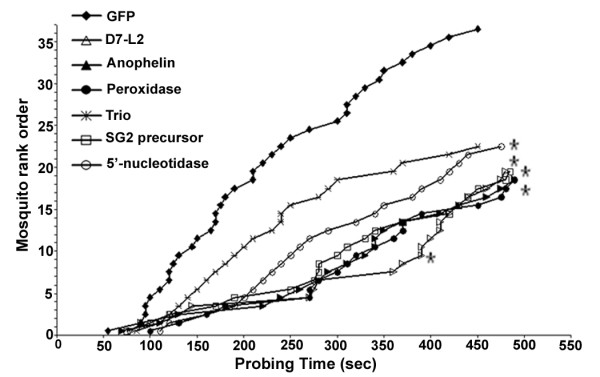
**Functional implication of salivary gland genes in the probing behavior of mosquitoes**. Probing time spent by gene silenced mosquitoes (depleted of *D7-L2*, *anophelin*, *peroxidase*, *trio*, *SG2 precursor *and *5' nucleotidase *transcripts), and GFP dsRNA injected control mosquitoes, from the insertion of mouthparts into the mouse skin and the first sign of blood within the midgut. Four days after dsRNA injection, individual mosquitoes were exposed to an anesthetized mouse for 500 seconds. Probing times were only measured for those mosquitoes that initiated ingestion of blood within the 500 seconds interval. GFP (n = 37), *D7-L2 *(n = 20; p = 0.002), *anophelin *(n = 19; p = 0.012), *peroxidase *(n = 19; p = 0.007), *trio *(n = 23; p = 0.7), *SG2 precursor *(n = 20; p = 0.02), *5' nucleotidase *(n = 23; p = 0.05), where n is the total number of mosquitoes tested for each gene and p value is determined by the Mann-Whitney test. Results from three independent sets of experiments are shown in the graph. The rank order (the cumulative number of mosquitoes from different replicas that completed their probing within this time-interval) was plotted against the time.

In a second set of assays we determined the effect of salivary gland transcript depletion on the mosquitoes probing time (Figure 3), as a measure of time, or interval, between the insertion of mouthparts into the host skin and the initiation of blood ingestion, according to methodology established in previous studies [9,11]. Of the six genes that were selected for probing time assays, depletion of *D7L2*, *anophelin*, *peroxidase *and *SG2 precursor *(Figure 2A) resulted in decreased blood feeding capacity and as well as increased probing time (Figure 3). These results strongly suggest a vital role in the blood-feeding process for these genes. Depletion of the *5' nucleotidase *resulted in a significantly longer probing time (Figure 3) but not a significantly altered blood-feeding capacity (Figure 2A). This indicates that blood-feeding capacity is not exclusively dependent on probing time.

The depletion of *TRIO *(p = 0.7) did not result in any significant changes either in blood-feeding behavior or probing time.

## Conclusions

The mosquito salivary glands play essential roles in blood-feeding and pathogen transmission. We have used a genome-wide microarray approach to characterize the *A. gambiae *salivary gland transcriptome, which corresponds to 4,978 genes (Figure 1), thereby extending earlier work based on cDNA/EST library clones [1,21,57]. Through the use of a full-genome microarray, we have obtained information on salivary gland transcript abundance and assessed the impact of blood feeding on salivary gland gene transcript abundance. The 93 transcripts showing significant differential abundance between the salivary glands of unfed and blood-fed female mosquitoes were mainly involved in metabolic, immunity, and oxidative stress-related processes (Figure 1D). It will be interesting to know whether the salivary gland expressed immune genes are involved in the interaction with, or killing of, the *Plasmodium *sporozoites. Interestingly, the majority of the secretory salivary gland-expressed transcripts displayed a lower abundance after blood-feeding, suggesting that their products may have been employed for protein synthesis during feeding and eventually degraded, or their transcription had been shut down. The lower blood-feeding capacity and increased probing time of mosquitoes upon silencing of the *D7L2*, *anophelin*, *peroxidase 5B*, *SG2 precursor*, and *5' nucleotidase *genes is consistent with their role as anti-hemostatic factors (Figure 2A and Figure 3) [1,62].

A better understanding of salivary gland gene expression and function can contribute to the development of malaria control strategies based on blocking the *Plasmodium *parasite in genetically modified mosquitoes or transmission blocking vaccines that could inhibit infection of the glands [66]. Furthermore, our studies indicate that the salivary glands of *Anopheles *mosquitoes express a variety of pharmacologically active substances that may be of value for designing novel anti-thrombotic or anti-platelet drugs for the treatment of cardiovascular disorders.

## Methods

### Rearing of *A. gambiae *mosquitoes

*A. gambiae *Keele strain mosquitoes were reared at 27°C in 70% humidity, and adults were maintained on a 10% sucrose solution on a 12-h light/dark cycle, according to standard rearing conditions [67].

### RNA isolation and quantitative real-time PCR (qRT-PCR)

RNA was extracted and quantified (in triplicate samples) from dissected salivary glands (~80-100) using an RNeasy kit (Qiagen, Valencia, California, USA) according to standard methodology [68]. The qRT-PCR assays were performed as previously described [68], and the ribosomal protein S7 gene was used for normalization of the cDNA templates. The gene silencing efficiency (see below) was calculated according to the standard E^ΔΔCt ^method [69].

### Microarray analysis

*A. gambiae *mosquitoes were allowed to feed for 20 min on Swiss Webster strain mice that had been anesthetized with a ketamine solution. For the microarray transcription analysis, approximately 100 salivary glands in triplicate were dissected 2 h after blood feeding (from both blood-fed and unfed mosquitoes). The blood-fed mosquitoes were not provided sugar solution for 2 hrs after blood-feeding, after which the salivary glands were dissected. The control unfed mosquitoes were treated in the same way. It should be noted that the triplicate assays were performed on different cohorts and generations. For the transcriptomic studies involving blood-fed regulated salivary gland gene expression, probes prepared from the RNA of salivary glands from blood-fed mosquitoes (labeled with Cy-5-dUTP fluorescent nucleotides) were co-hybridized with probes prepared from the RNA (labeled with Cy-3-dUTP fluorescent nucleotides) obtained from salivary glands of unfed mosquitoes. An Agilent custom-made 60-mer oligonucleotide microarray representing the full *A. gambiae *transcriptome was used for the assays. The Cy-5- and Cy-3-labeled probes were synthesized using the Low RNA Input Fluorescent Linear Amplification Kit from Agilent Technologies (Santa Clara, California, USA), and the slides were scanned using a GenePix 7000 autoloader scanner (Axon Instruments), as previously described in Dong *et al.*, 2006. Scanning and data analyses were performed as previously described, with a signal cut-off intensity of 100 to remove low-intensity/poorly hybridized spots from the analysis. Loc-Fit normalization (LOWESS) was performed independently for each data set. The TMEV software, available online (http://www.tm4.org/mev/), was then used for t-test analysis at a significance level of p < 0.05, to evaluate the variability between biological replicates. Only transcripts that had signal values above or below a log_2 _cutoff value of ± 0.75 were considered significantly regulated with blood-feeding and used for further analysis (Figure 1D). If a particular gene was not regulated in the same direction in all the three biological replicates, it was considered to be non-significantly regulated. The GEO-NCBI accession numbers are: GSM573605, GSM573606 and GSM573607.

For the determination of the global *A. gambiae female *salivary gland transcriptome, the microarray spot hybridization fluorescence intensities were used as an indicator of transcript abundance/expression level, since each spot contained a similar amount of nucleic acid. First, the spot fluorescence intensity values from the three replicate assays were averaged for each spot and then for each gene (some genes were represented by multiple spots), generating a list of transcripts that were expressed in the *A. gambiae *female salivary gland. Transcripts were then categorized in groups on the basis of their relative transcript abundance, as measured by the mean fluorescence value (at 635 nm) of each transcript. This analysis produced the following three groups: i) highly abundant transcripts (fluorescence intensity of 5,000 to the maximum threshold of 65,000), ii) moderately expressed transcripts (1,000 to 4,999) and iii) poorly expressed transcripts (100 to 999) (listed as three different Excel spreadsheets in Additional file 1). The average fluorescence values at both 635 and 532 nm from the three GPR files (Genepix) are listed in Additional file 1, for direct comparison of each transcript before and after blood-feeding. The lists of genes were then further sub-grouped into different functional groups according to their known functions (Figure 1). The microarray validation by independent quantitative real-time PCR (qRT-PCR) on some of the genes was performed (data not shown) as described in our earlier publications [68,70]. The validation data showed a high degree of correlation between the microarray and qRT-PCR values (Pearson correlation coefficient, p = 0.91; best-fit linear regression, R^2 ^= 0.84; slope of the regression line = 0.85).

### RNAi gene-silencing assays in adult mosquitoes

RNA interference (RNAi) assays in adult female mosquitoes were performed according to established RNAi methodology [71], using GFP dsRNA as the control. At least three independent RNAi-mediated gene-silencing assays were performed for each gene on different days, with two replicates each time; ~ 35-40 mosquitoes were injected for each replicate, and 25 randomly selected surviving mosquitoes from each replicate were used for further blood-feeding assays. It should be noted that the triplicate assays were conducted on different days, and the mosquitoes were from different cohorts/generations.

To silence the transcripts in the salivary glands, at least 8-9 times more dsRNA (~1.6-1.8 μg per mosquito) than normal (~0.2 μg) was injected [9] in order to obtain a better silencing efficiency in this body part, which is less accessible to diffusion of dsRNA from the hemolymph. The primer sequences of the selected genes and the GFP used to generate dsRNA are listed in Additional file 5. For gene silencing verification by qRT-PCR, ~40-50 mosquitoes were injected with dsRNA, and the salivary glands were dissected 4 days later. The ribosomal protein S7 gene was used for normalization of the cDNA templates (primer sequences are provided in Additional file 5). The fold difference in transcript abundance levels (the silencing efficiency) after silencing was calculated according to the standard E^ΔΔCt ^method [69]. The primers used for silencing verification are presented in Additional file 6.

### Blood-feeding assays

To study the blood-feeding propensity upon gene silencing, mosquitoes were exposed to anesthetized (with ketamine solution) Swiss Webster mice 4 days after the dsRNA injections. The mosquitoes were allowed to feed for 20 min, after which we scored the number of mosquitoes that had fed. Two mice were required for each feeding experiment (GFP control and experimental), and were used simultaneously by placing each mouse with its head over one cage and its tail over the other. Every 5 min, the positions of the mice were flipped (with minimum interruption) to ensure that the two mosquito sets drew blood from the identical body parts of the two mice. For statistical analysis of the gene silencing phenotypes (blood-feeding propensity) with respect to GFP control, a Mann Whitney test was used, as shown in our earlier publications [68,72]; the resulting p-values are given in Figure 2A and Additional file 4.

### Probing assays

Three days-old adult female mosquitoes were injected with dsRNA as described above and their probing time was assayed four days later. The mosquitoes were starved from sugar for 4-5 hours before conducting the probing time measurements as described in Lombardo *et al*., 2009. A single mosquito was transferred in a small cage and allowed to rest for 30 min before being offered an anesthetized (with ketamine solution) Swiss Webster mouse. Probing time is defined as the time taken from the initial insertion of the mouthparts in the skin until the initial engorgement of blood [9,11,73]. If the mosquito terminates the probing unsuccessfully and tries again elsewhere, the second probing time is progressively added to the first one until blood is ingested. The time between two probings (interprobing time), is not included. The assay was conducted for 500 seconds for each mosquito. Three independent sets of dsRNA injections for probing time were performed for each of the six genes and were compared to the probing time of GFP dsRNA injected control mosquitoes. The results from all replica assays were pooled and the rank order of the mosquitoes (the cumulative number of mosquito pooled from different replicas that completes their probing, at any particular time within the 500 seconds period) was plotted against time (seconds), as performed in earlier studies [9,11]. The data was then analyzed by the Mann-Whitney test for statistical significance as described in Lombardo *et al*., 2009. The total number of mosquitoes tested (n) and the resulting p-values for each silenced transcript and GFP dsRNA injected control are shown in Figure 3 legend.

## Abbreviations

GFP: green fluorescent protein; qRT-PCR: quantative real-time polymerase chain reaction; RNAi: RNA interference; OBP: odorant binding proteins; dsRNA: double-strand RNA.

## Authors' contributions

SD and AR conducted the microarray and gene silencing assays and SD contributed to the writing of the manuscript. YJC, AM and JGV performed preliminary and confirmatory analyses and assays and edited the manuscript. GD designed the experiments and the oligonucleotide microarrays and contributed to the writing of the manuscript. All authors read and approved the final manuscript.

## Supplementary Material

Additional file 1 **List of genes expressed in the female salivary gland of *A. gambiae*, along with their previous (ENSANGT) and recent (AGAP-R) transcript IDs**. Note that the recent AGAP-R IDs for some transcripts are missing in VectorBase and have therefore been left blank in column B. The fluorescent values at 635 nm (Cy-5 dye) and 532 nm (Cy-3 dye) for each gene are shown in column E and F respectively. The excel file has three sheets: Sheet 1 lists the genes that are highly expressed, with fluorescent values (at 635 nm) from 5,000 to the maximum threshold of 65,000, Sheet 2 lists the moderately expressed genes with fluorescent values (at 635 nm) between 1,000 to 4,999 and Sheet 3 lists the weakly expressed genes with fluorescent values (at 635 nm) between 100 to 999. The gene list is further sorted into different functional groups, according to the predicted function of the genes [CS: cytoskeletal and structural; CIR: circadian; CSR: chemosensory; PROT/DIG: proteolytic digestion; IMM: immunity; MET/ENZ: metabolism and enzymes; RED/STR: redox/stress; R/T/T: replication/transcription and translation; TRP: transport]. The list of genes in each group is further sorted into descending order of the fluorescent value at 635 nm (column E). Click here for file

Additional file 2 **List of genes that are regulated upon blood-feeding in the female *A. gambiae *salivary gland, along with their ENSANGT and more recent AGAP-R transcript IDs**. Transcripts lacking AGAP-R IDs are only indicated with the ENSANT transcript ID. Genes are only listed if they display significant differential regulation between fed and unfed mosquitoes, at a log_2 _fold ratio above or below the ± 0.75 threshold. Predicted functional groups classification for genes are also presented [CS: cytoskeletal and structural; CIR: circadian; CSR: chemosensory; PROT/DIG: proteolytic digestion; IMM: immunity; MET/ENZ: metabolism and enzymes; RED/STR: redox/stress; R/T/T: replication/transcription and translation; TRP: transport]. Click here for file

Additional file 3 **Gene silencing efficiency of the 10 selected genes**. The gene transcript abundance in GFP dsRNA treated mosquitoes was set to 1.0, and the corresponding percentage silencing was determined by qRT-PCR. The AgS7 gene was used for normalization of cDNA templates. The standard error values are shown. Click here for file

Additional file 4 **The blood-feeding propensity of mosquitoes in gene silenced mosquitoes**. Ten salivary gland expressed genes were individually silenced in mosquitoes, and the mosquitoes were allowed to feed on blood, 4 days after dsRNA injections. The percentages of mosquitoes that feed ("Fed") were scored and are shown along with their standard error values. The p-value from statistical analysis of Mann Whitney Test is also shown. Click here for file

Additional file 5 **Primers used for synthesis of dsRNAs for RNAi gene silencing assays**. The first 20 bases in bold correspond to the T7 polymerase promoter site. The transcript ID numbers (AGAP-RA from VectorBase) are also shown for each gene. The list also includes the sequences of GFP primers used for generating dsRNA and the *A. gambiae *S7 primer (used for standardization of cDNA templates). Click here for file

Additional file 6 **Primers used for verification of RNAi silencing**. The sense primers were newly designed (VERI Sense), and the antisense primers used were the same as those used for making the corresponding dsRNAs. The transcript ID numbers (AGAP-RA from VectorBase) are also shown for each gene. Click here for file
