# Supplementary material for: Transcriptomic and functional analysis of the Anopheles gambiae salivary gland in relation to blood feeding
Source: BMC Genomics. 2010 Oct 14;11:566. doi: 10.1186/1471-2164-11-566 (PMC3091715; doi:10.1186/1471-2164-11-566)
Supplement: Additional file 3 — Gene silencing efficiency of the 10 selected genes. The gene transcript abundance in GFP dsRNA treated mosquitoes was set to 1.0, and the corresponding percentage silencing was determined by qRT-PCR. The AgS7 gene was used for normalization of cDNA templates. The standard error values are shown. [file 1471-2164-11-566-S3.DOC]

**Transcriptomic and functional analysis of the *Anopheles gambiae* salivary gland in relation to blood feeding**

**Suchismita Das^1^ Andrea Radtke^1^, Young-Jun Choi^2^, Antonio M. Mendes^1, 3^, Jesus G. Valenzuela^4^ and George Dimopoulos^1, #^**

^1^W. Harry Feinstone Department of Molecular Microbiology and Immunology, Bloomberg School of Public Health, Johns Hopkins University, 615 N Wolfe Street, Baltimore, MD 21205-2179, USA.

^2^ Department of Pathobiological Sciences, University of Wisconsin-Madison, 1656 Linden Drive, Madison, WI 53706, USA.

^3^ Imperial College London, Division of Cell and Molecular Biology, Faculty of Natural Sciences, South Kensington Campus, London, United Kingdom.

^4^ Laboratory of Malaria and Vector Research, NIAID, National Institutes of Health, Rockville, Maryland 20852, USA.

Email addresses:

SD: [sudas@jhsph.edu](mailto:sudas@jhsph.edu)

AD: [aradtke@jhsph.edu](mailto:aradtke@jhsph.edu)

YJC: [ychoi24@wisc.edu](mailto:ychoi24@wisc.edu)

YM: [antonio.mendes@imperial.ac.uk](mailto:antonio.mendes@imperial.ac.uk)

JGV: [jvalenzuela@niaid.nih.gov](mailto:jvalenzuela@niaid.nih.gov)

GD: [gdimopou@jhsph.edu](mailto:gdimopou@jhsph.edu)

**Additional file 3**

**Gene silencing efficiency of the 10 selected genes.** The gene transcript abundance in GFP dsRNA treated mosquitoes was set to 1.0, and the corresponding percentage silencing was determined by qRT-PCR. The AgS7 gene was used for normalization of cDNA templates. The standard error values are shown.

| **Gene name** | **% of silencing**  **with respect to GFP control** | **Standard error** |
| --- | --- | --- |
| *D7 L1 long protein* | 40 | 0.05 |
| *D7 L2 long protein* | 46 | 0.09 |
| *Anophelin* | 43 | 0.07 |
| *SG Peroxidase 5B* | 38 | 0.11 |
| *Trio* | 34 | 0.06 |
| *5’ Nucleotidase* | 38 | 0.11 |
| *Salivary mucin* | 34 | 0.12 |
| *30 kD protein* | 19 | 0.07 |
| *Salivary lipase* | 29 | 0.13 |
| *SG2 precursor* | 29 | 0.08 |
