# Supplementary material for: Transcriptomic and functional analysis of the Anopheles gambiae salivary gland in relation to blood feeding
Source: BMC Genomics. 2010 Oct 14;11:566. doi: 10.1186/1471-2164-11-566 (PMC3091715; doi:10.1186/1471-2164-11-566)
Supplement: Additional file 4 — The blood-feeding propensity of mosquitoes in gene silenced mosquitoes. Ten salivary gland expressed genes were individually silenced in mosquitoes, and the mosquitoes were allowed to feed on blood, 4 days after dsRNA injections. The percentages of mosquitoes that feed ("Fed") were scored and are shown along with their standard error values. The p-value from statistical analysis of Mann Whitney Test is also shown. [file 1471-2164-11-566-S4.DOC]

**Transcriptomic and functional analysis of the *Anopheles gambiae* salivary gland in relation to blood feeding**

**Suchismita Das^1^ Andrea Radtke^1^, Young-Jun Choi^2^, Antonio M. Mendes^1, 3^, Jesus G. Valenzuela^4^ and George Dimopoulos^1, #^**

^1^W. Harry Feinstone Department of Molecular Microbiology and Immunology, Bloomberg School of Public Health, Johns Hopkins University, 615 N Wolfe Street, Baltimore, MD 21205-2179, USA.

^2^ Department of Pathobiological Sciences, University of Wisconsin-Madison, 1656 Linden Drive, Madison, WI 53706, USA.

^3^ Imperial College London, Division of Cell and Molecular Biology, Faculty of Natural Sciences, South Kensington Campus, London, United Kingdom.

^4^ Laboratory of Malaria and Vector Research, NIAID, National Institutes of Health, Rockville, Maryland 20852, USA.

Email addresses:

SD: [sudas@jhsph.edu](mailto:sudas@jhsph.edu)

AD: [aradtke@jhsph.edu](mailto:aradtke@jhsph.edu)

YJC: [ychoi24@wisc.edu](mailto:ychoi24@wisc.edu)

YM: [antonio.mendes@imperial.ac.uk](mailto:antonio.mendes@imperial.ac.uk)

JGV: [jvalenzuela@niaid.nih.gov](mailto:jvalenzuela@niaid.nih.gov)

GD: [gdimopou@jhsph.edu](mailto:gdimopou@jhsph.edu)

**Additional file 4:**

**The blood-feeding propensity of mosquitoes in silenced mosquitoes.** Ten salivary gland genes were individually silenced in mosquitoes, and the mosquitoes were allowed to feed on blood, 4 days after dsRNA injections. The percentages of mosquitoes that feed (“Fed”) were scored and are shown along with their standard error values. The p- value from statistical analysis of Mann Whitney Test is also shown.

| **Gene name** | **Percentage of “Fed” mosquitoes** | **Standard**  **error** | **p-value**  **(Mann**  **Whitney test)** | **Significant /**  **Non-significant**  **(S/NS)** |
| --- | --- | --- | --- | --- |
| *GFP* | 47.5 | 3.1 | N/A | N/A |
| *D7 L1 long protein* | 39.5 | 1.9 | 0.49 | NS |
| *D7 L2 long protein* | 31.3 | 1.4 | 0.03 | S |
| *Anophelin* | 28.8 | 2.4 | 0.03 | S |
| *SG Peroxidase 5B* | 32.5 | 2.2 | 0.04 | S |
| *Trio* | 49.5 | 3.8 | 0.93 | NS |
| *5’ Nucleotidase* | 37 | 2.8 | 0.07 | NS |
| *Salivary mucin* | 44.5 | 1.9 | 0.93 | NS |
| *30 kD protein* | 43 | 2.8 | 0.10 | NS |
| *Salivary lipase* | 35.5 | 3.2 | 0.09 | NS |
| *SG2 precursor* | 29.3 | 1.6 | 0.03 | S |
