# Supplementary material for: Transcriptomic and functional analysis of the Anopheles gambiae salivary gland in relation to blood feeding
Source: BMC Genomics. 2010 Oct 14;11:566. doi: 10.1186/1471-2164-11-566 (PMC3091715; doi:10.1186/1471-2164-11-566)
Supplement: Additional file 5 — Primers used for synthesis of dsRNAs for RNAi gene silencing assays. The first 20 bases in bold correspond to the T7 polymerase promoter site. The transcript ID numbers (AGAP-RA from VectorBase) are also shown for each gene. The list also includes the sequences of GFP primers used for generating dsRNA and the A. gambiae S7 primer (used for standardization of cDNA templates). [file 1471-2164-11-566-S5.DOC]

**Transcriptomic and functional analysis of the *Anopheles gambiae* salivary gland in relation to blood feeding**

**Suchismita Das^1^ Andrea Radtke^1^, Young-Jun Choi^2^, Antonio M. Mendes^1, 3^, Jesus G. Valenzuela^4^ and George Dimopoulos^1, #^**

^1^W. Harry Feinstone Department of Molecular Microbiology and Immunology, Bloomberg School of Public Health, Johns Hopkins University, 615 N Wolfe Street, Baltimore, MD 21205-2179, USA.

^2^ Department of Pathobiological Sciences, University of Wisconsin-Madison, 1656 Linden Drive, Madison, WI 53706, USA.

^3^ Imperial College London, Division of Cell and Molecular Biology, Faculty of Natural Sciences, South Kensington Campus, London, United Kingdom.

^4^ Laboratory of Malaria and Vector Research, NIAID, National Institutes of Health, Rockville, Maryland 20852, USA.

Email addresses:

SD: [sudas@jhsph.edu](mailto:sudas@jhsph.edu)

AD: [aradtke@jhsph.edu](mailto:aradtke@jhsph.edu)

YJC: [ychoi24@wisc.edu](mailto:ychoi24@wisc.edu)

YM: [antonio.mendes@imperial.ac.uk](mailto:antonio.mendes@imperial.ac.uk)

JGV: [jvalenzuela@niaid.nih.gov](mailto:jvalenzuela@niaid.nih.gov)

GD: [gdimopou@jhsph.edu](mailto:gdimopou@jhsph.edu)

**Additional file 5:**

**Primers used for synthesis of dsRNAs for RNAi gene silencing assays.**

The first 20 bases in bold correspond to the T7 polymerase promoter site. The transcript ID numbers (AGAP-RA from VectorBase) are also shown for each gene. The list also includes the primer sequences of GFP primers used for generating dsRNA and the *A. gambiae* S7 primer (used for standardization of cDNA templates).

1. D7 L1 long protein: AGAP008278-RA

D7 L1 Forward: **TAATACGACTCACTATAGGG**GATCTTTCCCGCAAACTGTA

D7 L1 Reverse: **TAATACGACTCACTATAGGG**TCTGGCACACTCTTCAATGG

2. D7 L2 long protein: AGAP008279-RA

D7 L2 Forward: **TAATACGACTCACTATAGGG**TACCATGGTACGGTTGAGGGA

D7 L2 Reverse: **TAATACGACTCACTATAGGG**ATGGAATTCTTGGAAGCTTCA

3. Anophelin: AGAP008004-RA

Anophelin Forward: **TAATACGACTCACTATAGGG**GTGGTGGTTGTGCAGAGTGC

Anophelin Reverse: **TAATACGACTCACTATAGGG**GAGCAGATGCTTGTTCGTCA

4. SG Peroxidase 5B: **AGAP010735-RA**

SG Peroxidase Forward: **TAATACGACTCACTATAGGG**GAGCTAATGCGGATCGCTAC

SG Peroxidase Reverse: **TAATACGACTCACTATAGGG**GTTGGGCTCCAGTTTTTGAA

5. Trio: **AGAP001374-RA**

Trio Forward: **TAATACGACTCACTATAGGG**AGTATGTACGACCTGATCGGCC

Trio Reverse: **TAATACGACTCACTATAGGG**TGAATTTGGCCATCAGATCA

6. 5’ Nucleotidase: AGAP011026-RA

5’ Nucleotidase Forward: **TAATACGACTCACTATAGGG**CGTCGTGGTACCGTCCTATT

5’ Nucleotidase Reverse: **TAATACGACTCACTATAGGG**TACCGTTGGTGTGGTTCTCA

7. Salivary mucin (SM): AGAP001192-RA

SM Forward: **TAATACGACTCACTATAGGG**TAGATCCTCCCACTACTACGG

SM Reverse: **TAATACGACTCACTATAGGG**ACTGGTACGAGGGCAGCTTTCA

8. 30 kD protein: AGAP009974-RA

30 kD Forward: **TAATACGACTCACTATAGGGT**GATCAAGAATCATCTACTGA

30 kD Reverse: **TAATACGACTCACTATAGGG**ACTGCATCATCGCTTCCTGCA

9. Salivary lipase (SL): AGAP005822-RA

SL Forward: **TAATACGACTCACTATAGGG**TCCTGCTCGACTACGTCAACA

SL Reverse: **TAATACGACTCACTATAGGG** TCGTAGCCTTCCAGCCGCACCT

10. SG2 precursor: AGAP006506-RA

SG2 Forward: **TAATACGACTCACTATAGGGT**GGCGGTGGCGGTTACTTCAT

SG2 Reverse: **TAATACGACTCACTATAGGG**ATGGACGAGAACGCATCGGTA

11. GFP:

GFP Forward: **TAATACGACTCACTATAGGG**ATGGTGAGCAAGGGCGAGGAGCTGT

GFP Reverse: **TAATACGACTCACTATAGGG**TTACTTGTACAGCTCGTCCATGCCG

12. *A. gambiae* S7 gene primer sequences (for standardization of cDNA templates):

S7 Forward: TCCTGGAGCTGGAGATGAAC.

S7 Reverse: GACGGGTCTGTACCTTCTGG.
