# Supplementary material for: Transcriptomic and functional analysis of the Anopheles gambiae salivary gland in relation to blood feeding
Source: BMC Genomics. 2010 Oct 14;11:566. doi: 10.1186/1471-2164-11-566 (PMC3091715; doi:10.1186/1471-2164-11-566)
Supplement: Additional file 6 — Primers used for verification of RNAi silencing. The sense primers were newly designed (VERI Sense), and the antisense primers used were the same as those used for making the corresponding dsRNAs. The transcript ID numbers (AGAP-RA from VectorBase) are also shown for each gene. [file 1471-2164-11-566-S6.DOC]

**Transcriptomic and functional analysis of the *Anopheles gambiae* salivary gland in relation to blood feeding**

**Suchismita Das^1^ Andrea Radtke^1^, Young-Jun Choi^2^, Antonio M. Mendes^1, 3^, Jesus G. Valenzuela^4^ and George Dimopoulos^1, #^**

^1^W. Harry Feinstone Department of Molecular Microbiology and Immunology, Bloomberg School of Public Health, Johns Hopkins University, 615 N Wolfe Street, Baltimore, MD 21205-2179, USA.

^2^ Department of Pathobiological Sciences, University of Wisconsin-Madison, 1656 Linden Drive, Madison, WI 53706, USA.

^3^ Imperial College London, Division of Cell and Molecular Biology, Faculty of Natural Sciences, South Kensington Campus, London, United Kingdom.

^4^ Laboratory of Malaria and Vector Research, NIAID, National Institutes of Health, Rockville, Maryland 20852, USA.

# Corresponding author

Email addresses:

SD: [sudas@jhsph.edu](mailto:sudas@jhsph.edu)

AD: [aradtke@jhsph.edu](mailto:aradtke@jhsph.edu)

YJC: [ychoi24@wisc.edu](mailto:ychoi24@wisc.edu)

YM: [antonio.mendes@imperial.ac.uk](mailto:antonio.mendes@imperial.ac.uk)

JGV: [jvalenzuela@niaid.nih.gov](mailto:jvalenzuela@niaid.nih.gov)

GD: [gdimopou@jhsph.edu](mailto:gdimopou@jhsph.edu)

**Additional file 6:**

**Primers used for verification of RNAi silencing.**

The sense primers were newly designed (VERI Sense), and the antisense primers used were the same as those used for making the corresponding dsRNAs. The transcript ID numbers (AGAP-RA from VectorBase) are also shown for each gene.

1. D7 L1 long protein: AGAP008278-RA.

D7 L1 Veri Forward: ACACATTTAAGCCCGAAACG

D7 L1 Reverse: **TAATACGACTCACTATAGGG**TCTGGCACACTCTTCAATGG

2. D7 L2 long protein: AGAP008279-RA.

D7 L2 Veri Forward: TGCGCTGTATAACGCCTATCT

D7 L2 Reverse: **TAATACGACTCACTATAGGG**ATGGAATTCTTGGAAGCTTCA

3. Anophelin: AGAP008004-RA

Anophelin Veri Forward: TCGTGTTAGCCTTCCTGTGC

Anophelin Reverse: **TAATACGACTCACTATAGGG**GAGCAGATGCTTGTTCGTCA

4. SG Peroxidase 5B: **AGAP010735-RA**

SG Peroxidase Veri Forward: TAGTAACAGTGCAGTACCGT

SG Peroxidase Reverse: **TAATACGACTCACTATAGGG**GTTGGGCTCCAGTTTTTGAA

5. Trio: **AGAP001374-RA**

Trio Veri Forward: TATCGCAGTACGCTTGCATC

Trio Reverse: **TAATACGACTCACTATAGGG**TGAATTTGGCCATCAGATCA

6. 5’ Nucleotidase: AGAP011026-RA

5’ Nucleotidase Veri Forward: AGACTGCGCTTAAGAAGGACCA

5’ Nucleotidase Reverse: **TAATACGACTCACTATAGGG**TACCGTTGGTGTGGTTCTCA

7. Salivary mucin (SM): AGAP001192-RA

SM Veri Forward: TGTCCTGCTCGCAGTCACCAGT

SM Reverse: **TAATACGACTCACTATAGGG**ACTGGTACGAGGGCAGCTTTCA

8. 30 kD protein: AGAP009974-RA

30 Kd Veri Forward: TATGCCTTGTGCTAATCGTAT

30 kD Reverse: **TAATACGACTCACTATAGGG**ACTGCATCATCGCTTCCTGCA

9. Salivary lipase: AGAP005822-RA

SL Veri Forward: TGCACCAACTGGTGCGTAATGT

SL Reverse: **TAATACGACTCACTATAGGG** TCGTAGCCTTCCAGCCGCACCT

10. SG2 precursor: AGAP006506-RA

SG2 Veri Forward: AGTCGCGCTGGTGGTGGTCGT

SG2 reverse: **TAATACGACTCACTATAGGG**ATGGACGAGAACGCATCGGTA
